# P93 UK TB medication shortages—hard to swallow pills

**DOI:** 10.1093/jacamr/dlaf230.100

**Published:** 2025-12-04

**Authors:** Leena Begum, Andrew Brush

**Affiliations:** Sandwell and West Birmingham NHS Trust, Smethwick, England; Sandwell and West Birmingham NHS Trust, Smethwick, England

## Abstract

**Background:**

TB is a potentially fatal infection with a rising incidence in the UK. Curative treatment is available but requires prolonged courses of multiple medications (particularly during the initial phase treatment). Adherence to therapy is critical to success. Fixed-dose combination (FDC) products combine multiple medications into a single pill to maximize adherence and are used first-line. However, shortages of FDCs have plagued UK supply chains leading to a National Patient Safety Alert in July 2025. Supplying individual component medications in small amounts (1 month maximum) poses a risk to adherence and optimal outcomes. This is particularly concerning as many TB patients already have risk factors for poor adherence.

**Objectives:**

(i) Quantify impact of FDC shortages on pill burden and quantities of medication supplied by Sandwell and West Birmingham NHS Trust from 1^st^ July 2025. (ii) Contrast pill burden of dispensed therapy with an optimal FDC based regimen.

**Methods:**

Dispensing records held within the hospital pharmacy computer system were accessed for all patients receiving rifampicin or a rifampicin containing medication between 1 July 2025 and 17 Sept 2025 (all available data). Patients not being treated for TB were excluded. The details of the medication dispensed (product, strength, dose, quantity) were recorded in Microsoft Excel and pill burden calculated. A pharmacist used hospital records to determine the appropriate first-line FDC regimen and calculate the pill burden of this hypothetical treatment.

**Results:**

We identified 94 relevant prescriptions for 62 patients. Of these, 32 patients were still in the initial phase of TB treatment during the shortage. All 32 patients received multi-product regimens instead of the first-line rifampicin with ethambutol, isoniazid and pyrazinamide FDC. However, one received partial treatment with the first-line FDC and 13 received a multi-product regimen which included a rifampicin with isoniazid FDC. The median pill burden across all prescriptions was 7 (IQR 4.75–11). The median pill burden of optimal regimen was 3 (IQR 2–5). The median supply duration was 28 days (IQR 28–56 days) but with significant outliers (min 7 days, max 168 days). Patients did not always receive the same regimen for the entire phase of treatment; 18 had a different combination of products for subsequent prescriptions.

**Conclusions:**

Shortages of FDCs have more than doubled median pill burden for our patients. This is despite mitigation with unlicensed imports of rifampicin with isoniazid FDCs. Patients are required to reattend more frequently than usual to collect further supplies. Furthermore, there is no guarantee that a consistent combination of medication will be provided from one prescription to the next. These factors all contribute to an increased risk of poor adherence which is associated with treatment failure, onward transmission and increased drug resistance. Robust supply chains are critical to effective TB treatment.

